# Controversies in treating febrile infantile urinary tract infection caused by extended-spectrum beta-lactamase producing Enterobacteriaceae: an international multi-centre survey

**DOI:** 10.1007/s00467-025-06700-w

**Published:** 2025-02-04

**Authors:** Sze Wa Wong, Kjell Tullus, Yu Hin Eugene Chan

**Affiliations:** 1 Paediatric Nephrology Centre, Hong Kong Children’s Hospital, Kowloon Bay, Hong Kong SAR China; 2 Department of Paediatric Nephrology, Great Ormond Street Hospital for Children NHS Trust, London, UK; 3 Department of Paediatrics, The Chinese University of Hong Kong, Shatin, Hong Kong SAR China

**Keywords:** Antibiotics, ESBL, Enterobacteriaceae, Infant, Urinary tract infection

## Abstract

**Background:**

There is a lack of consensus in treating infants with extended-spectrum beta-lactamase producing Enterobacteriaceae (ESBL-E) urinary tract infection (UTI) who demonstrate good clinical response to initial antibiotics within 48 h.

**Methods:**

We conducted an international survey among paediatric nephrologists and fellows in training using a web-based questionnaire.

**Results:**

A total of 232 centres across 77 countries participated in the survey. Second- or third-generation cephalosporins were the initial antibiotic of choice upon presentation in 63.8% of the centres. If the ESBL-E isolated from urine culture demonstrated in vitro susceptibility, 81.0% of respondents would continue the initial oral antibiotics. In contrast, there was considerable practice variation in the presence of in vitro resistance to the initial oral antibiotic. 19.0% would switch to a carbapenem group antibiotic, while 49.6% would change to a non-carbapenem antibiotic according to the sensitivity profiles. 22.8% would continue initial antibiotics based on satisfactory clinical response. The remaining 8.6% would choose other options. Similar emphasis on in vitro susceptibility result for the treatment was observed among centres who treated patients with intravenous antibiotics at UTI presentation. In the presence of a UTI with an ESBL-E, 50.0% centres would perform additional radiological investigations, and 61.2% would offer antibiotic prophylaxis to prevent further UTIs.

**Conclusion:**

There are significant variations in the management of UTI caused by ESBL-E bacteria between centres. In vitro susceptibility to the antibiotics remains an important management consideration. Antibiotics from the non-carbapenem groups seem to be the preferred option. Further studies are required to identify the optimal treatment regimen in this patient population.

**Graphical abstract:**

**Figure:**
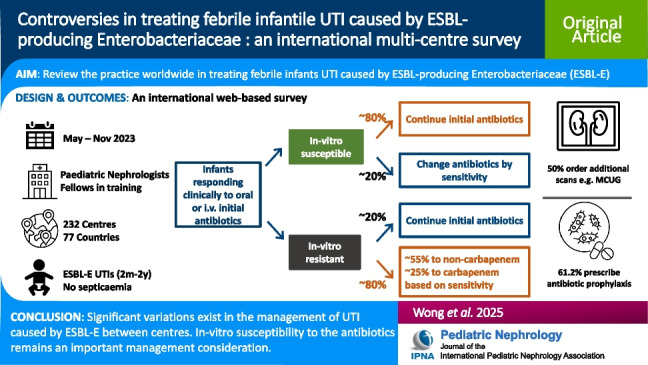
A higher resolution version of the Graphical abstract is available as Supplementary information

**Supplementary Information:**

The online version contains supplementary material available at 10.1007/s00467-025-06700-w.

## Introduction

Urinary tract infection (UTI) is one of the most common paediatric infections. While most cases are mild and respond well to treatment, serious life-threatening complications can occur, including severe sepsis and septic shock. While the causal relationship between UTI, kidney scarring and the development of chronic kidney disease (CKD) remains debatable, UTI can lead to a more rapid decline in residual kidney function in patients with pre-existing CKD [1].

Infantile UTIs are often caused by Enterobacteriaceae, such as *Escherichia coli* and *Klebsiella pneumoniae*, making beta-lactams and cephalosporins the recommended initial antibiotic of choice [2]. Historically, intravenous injection was the preferred route of treatment [3]. However, comparable clinical efficacy has been observed with oral therapy, which has now become the standard of care in many centres worldwide [4–6]. There has also been a considerable shift in follow-up imaging policies over the years, with ultrasonography now being the only first-line investigation required for most patients [7–9].

UTI caused by extended-spectrum beta-lactamase-producing Enterobacteriaceae (ESBL-E) has become more prevalent in recent decades [10, 11]. Bacteria that harbour this enzyme have extensive hydrolysis capabilities against a broad spectrum of beta-lactams and cephalosporins, posing significant management challenges [11, 12]. Carbapenem groups, which are available only as intravenous preparations, are the recommended therapy in ESBL-E infections, since treatment failures have been reported with other susceptible antibiotics [13, 14]. In a multi-centre observational study, Paterson et al. identified 32 patients with bacteraemia due to ESBL-E from various sources of infection including UTI. While 28 of these patients had ESBL-E strains susceptible to third-generation cephalosporins, 54% experienced treatment failure, defined as persistent fever or bacterial growth after 48 h of treatment [13]. Additionally, the 14-day mortality rate was significantly higher in patients treated with non-carbapenem group antibiotics, even when the bacteria were susceptible to these agents, compared to those treated with carbapenems [14]. This dissociation between in vitro activities and in vivo response has led many centres to prefer carbapenem group antibiotics. However, these studies only recruited patients with bacteraemia, leaving the response in patients without bacteraemia poorly defined. The practice of exclusively using intravenous (IV) antibiotic has placed significant burdens on patients, carers and health care workers, resulting in prolonged hospitalization, challenges in obtaining vascular access and increased parental anxiety [11]. Furthermore, the use of a carbapenem is associated with the emergence of carbapenem-resistant strains in the community [12]. Recently, treatment successes with non-carbapenem groups have also been reported in children with UTI caused by ESBL-E bacteria, observed in cases where the bacteria were either susceptible or resistant to the prescribed antibiotics [15–20].

These findings suggest that alternative treatment approaches should be considered for stable infants with febrile UTI who demonstrate satisfactory clinical response to initial antibiotic treatment. In view of the controversial practices in this specific patient population, our study aims to review the current practices worldwide and identify future research questions which can improve evidence-based management of UTI.

## Methods

We conducted an international survey in English on febrile UTI caused by ESBL-E bacteria in infants between 2 months and 2 years old through a web-based questionnaire using SurveyMonkey. Paediatric nephrologists and fellows in training were invited to participate via the International Paediatric Nephrology Association, European Society of Paediatric Nephrology, Paediatric Nephrology Information List (PEDNEPH, University of Chicago) and the author’s research collaborative network [21–23]. The surveying period ran from 1st May to 1st November 2023.

The questionnaire focused on three main themes: (1) general management of UTI, (2) management considerations in UTI caused by ESBL-E bacteria and (3) corresponding follow-up care. Since most paediatricians or paediatric nephrologists would typically treat UTI caused by ESBL-E bacteria with a carbapenem for patients who had septicaemia or did not respond to initial treatment, the second theme focused on the management of infants without septicaemia who showed satisfactory response to the initial antibiotic. Clinical response was defined as defervescence within 48–72 h of treatment. For this study, antibiotics were categorized into carbapenem and non-carbapenem groups. Carbapenems included meropenem and ertapenem. The non-carbapenems encompassed penicillin with beta-lactamase inhibitors, aminoglycosides, fluroquinolones and other groups specified by respondents, such as nitrofurantoin or co-trimoxazole.

The survey consisted of 15 questions, three of which had sub-questions. The questions are detailed in Supplemental Form 1. Response types included single choice questions (*n* = 8), multiple-choice questions (*n* = 3), Yes or No questions (*n* = 3) and scoring questions (*n* = 7) using a grading scale of 0 to 10 (10 = strongly agree, 5 = neutral, 0 = strongly disagree). An option of free text entry was available in nine questions. Respondents were able to review and change their answers before submission. The survey was conducted on a voluntary basis, and no incentives were offered. Data collected were fully anonymized and stored in the SurveyMonkey database till 09th November 2023. According to local policies in the Great Ormond Street Hospital for Children and Hong Kong Children’s Hospital, institutional review board approval for conducting a survey is not required.

## Statistical analysis

The IBM SPSS statistics version 26 software was used for statistical analysis. Descriptive statistics were used for demographic data as appropriate. Sub-group analysis was performed according to regions and socioeconomic status, as defined by the United Nations. Countries with a high or very high Human Development Index (HDI) were defined as developed countries, while those below were defined as developing countries. Data were compared between subgroups using Freeman–Halton extension of Fisher's exact test or chi-square test for the categorical variables, as appropriate. A *p*-value less than 0.05 in two tails was treated as significant in all tests.

## Results

### Demographic distribution of respondents

We received responses from 232 centres across 77 countries. Only one response was received from each centre. Among the participating centres, 37.9% (*n* = 88), 25.9% (*n* = 60), 14.7% (*n* = 34), 9.9% (*n* = 23), 7.3% (*n* = 17), 3.4% (*n* = 8) and 0.8% (*n* = 2) were from Europe, Asia, Middle East, South America, North America, Africa and Oceania, respectively (Supplemental Table 1).

### General management of UTI

While a majority of respondents (60.3%, *n* = 140) treated infantile UTI in accordance with institutional/local guidelines on UTI, 38.8% (*n* = 90) followed guidelines published by the American Academy of Paediatrics (AAP), and 26.7% (*n* = 62) adhered to those from the National Institute for Health and Care Excellence (NICE).

Cephalosporins were the preferred initial antibiotics, with third-generation cephalosporins used by 41.4% (*n* = 96) of respondents and second-generation by 22.4% (*n* = 52). In Europe, Asia, the Middle East and Africa, third-generation cephalosporins were favoured, while a comparable use of second and third generations was noted in North and South America. The preference for oral (*n* = 118, 50.9%) versus intravenous (*n* = 114, 49.1%) routes for the initial antibiotic administration was similar. Among centres that preferred the oral route, intravenous antibiotic administration would be considered in cases with severe sepsis (*n* = 108/118, 91.5%), history of underlying urological anomalies (*n* = 55/118, 46.6%) and in infants less than 12 months (*n* = 30/118, 25.4%). When clinical improvement was observed, 87.7% (*n* = 100/114) of respondents who started with IV antibiotics would switch to an oral antibiotic based on sensitivity patterns. In children with UTIs without septicaemia, most centres (77.1%, *n* = 179) preferred a duration of antibiotic treatment of 7 to 10 days. Importantly, an antibiotic targeting ESBL-E would be prescribed for patients with a previous history of UTI due to ESBL-E bacteria (*n* = 158, 68.1%) and those with congenital anomalies, e.g. vesicoureteral reflux (VUR) (*n* = 135, 58.2%).

### Prevalence of UTI caused by ESBL-E bacteria

The estimated prevalence of UTI caused by ESBL-E bacteria was ≤ 10%, 11–20% and > 20% in 48.2% (*n* = 109/226) and 34.1% (*n* = 77/226) and 17.7% (*n* = 40/226) of the centres, respectively (Table 1). Centres in Asia, the Middle East and Africa reported a significantly higher estimated prevalence (> 20%) of ESBL-E bacteria (*p* < 0.01) compared to centres in other regions. The choice of initial antibiotics correlated with the estimated prevalence of ESBL-E. Third-generation cephalosporins were more commonly used in centres reporting a high estimated prevalence (*n* = 26/40, 65.0%). In contrast, their use was lower in centres with a lower estimated prevalence: 11–20% (*n* = 34/77, 44.2%) and < 10% (*n* = 34/109, 31.2%). Conversely, penicillin with beta-lactamase inhibitors were more frequently utilized in centres with lower estimated ESBL prevalence: < 10% (*n* = 31/109, 28.4%), 11–20% (*n* = 11/77, 14.3%), and > 20% (*n* = 3/40, 7.5%) (*p* = 0.018) (Table 2).

**Table 1:** Estimated prevalence of ESBL-E UTI among centres from different continents

		Europe (*n* = 86)		North America (*n* = 17)		South America (*n* = 22)		Asia (*n* = 60)		Middle East (*n* = 33)		Africa (*n* = 6)		Australia (*n* = 2)	
Prevalence	≤ 10%	63	73.3%	10	58.8%	9	40.9%	23	38.3%	3	9.1%	1	16.7%	0	0
	11–20%	18	20.9%	5	29.4%	10	45.5%	22	36.7%	19	57.6%	2	33.3%	1	50.0%
	> 20%	5	5.8%	2	11.8%	3	13.6%	15	25.0%	11	33.3%	3	50.0%	1	50.0%

**Table 2:** Initial antibiotics in centres with different estimated ESBL-E prevalence

	Estimated prevalence						
Antibiotic choice	≤ 10% (*n* = 109)		11–20% (*n* = 77)		> 20% (*n* = 40)		*p*-value
Second generation cephalosporin	24	22.0%	21	27.3%	6	15.0%	0.018
Third generation cephalosporin	34	31.2%	34	44.2%	26	65.0%	
Penicillin with beta-lactamase inhibitor	31	28.4%	11	14.3%	3	7.5%	
Aminoglycoside	9	8.3%	7	9.1%	1	2.5%	
Carbapenem	3	2.8%	1	1.3%	1	2.5%	
Others	8	7.3%	3	3.9%	3	7.5%	

### Further antibiotic treatment of children with UTI due to ESBL-E bacteria

We evaluated the continued antibiotic treatment of children with UTI due to ESBL-E bacteria who had clinically responded to the empirical treatment. This evaluation considered four scenarios based on the route of administration (oral versus intravenous) and in vitro sensitivity (susceptible versus resistant) to the initial antibiotic.

If the patient was initially treated with an oral antibiotic and the urine culture demonstrated in vitro sensitivity, most respondents would continue the initial antibiotic regimen (*n* = 188/232, 81.0%), and 30.3% of them (*n* = 57/188) would extend the treatment duration. Only 8.6% (*n* = 20) and 4.7% (*n* = 11) of the centres would change to a susceptible antibiotic from the non-carbapenem group (oral or intravenous) or step up to intravenous carbapenems. The remaining 5.6% (*n* = 13) would choose other options. In contrast, if the ESBL-E organisms isolated from the urine culture showed in vitro resistance to the initial oral antibiotic, 49.6% (*n* = 115) of the respondents would change the treatment to another drug from the non-carbapenem groups (oral or intravenous) and 91.9% (*n* = 44) would step up to an intravenous carbapenem, according to the sensitivity results. Notably, 22.8% of the (*n* = 53) respondents would, despite in vitro resistance, continue the initial oral antibiotics in view of the satisfactory clinical response. The remaining 8.6% (*n* = 20) would choose other options.

**Fig. 1:**
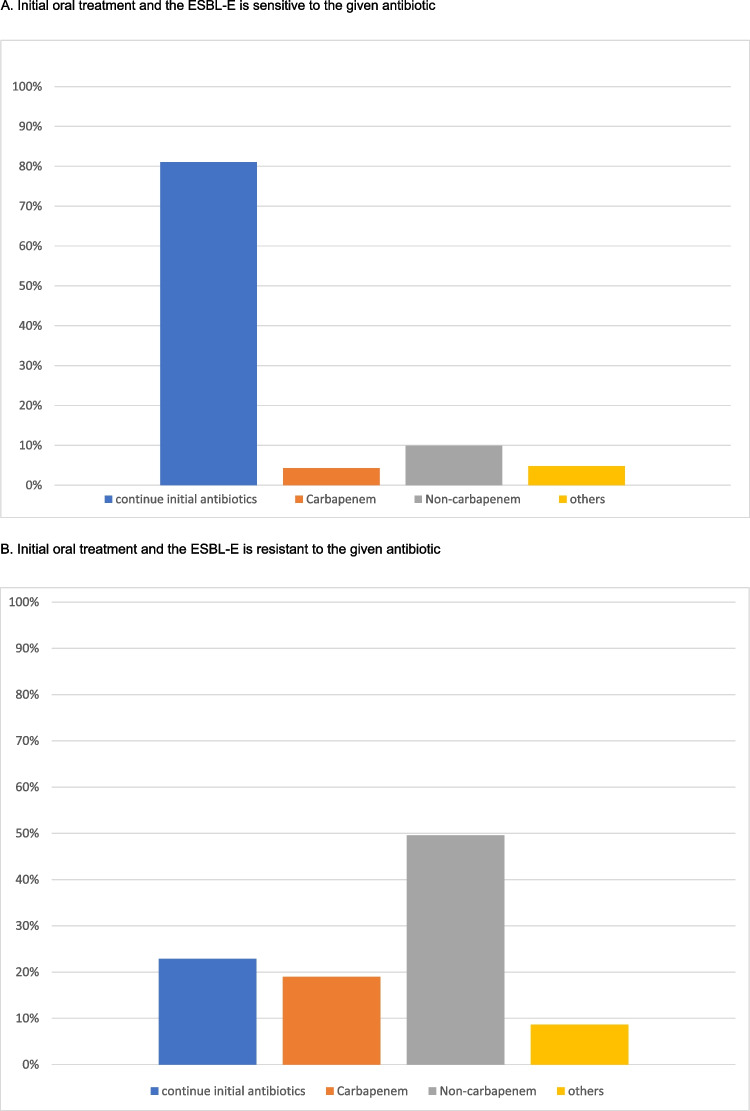

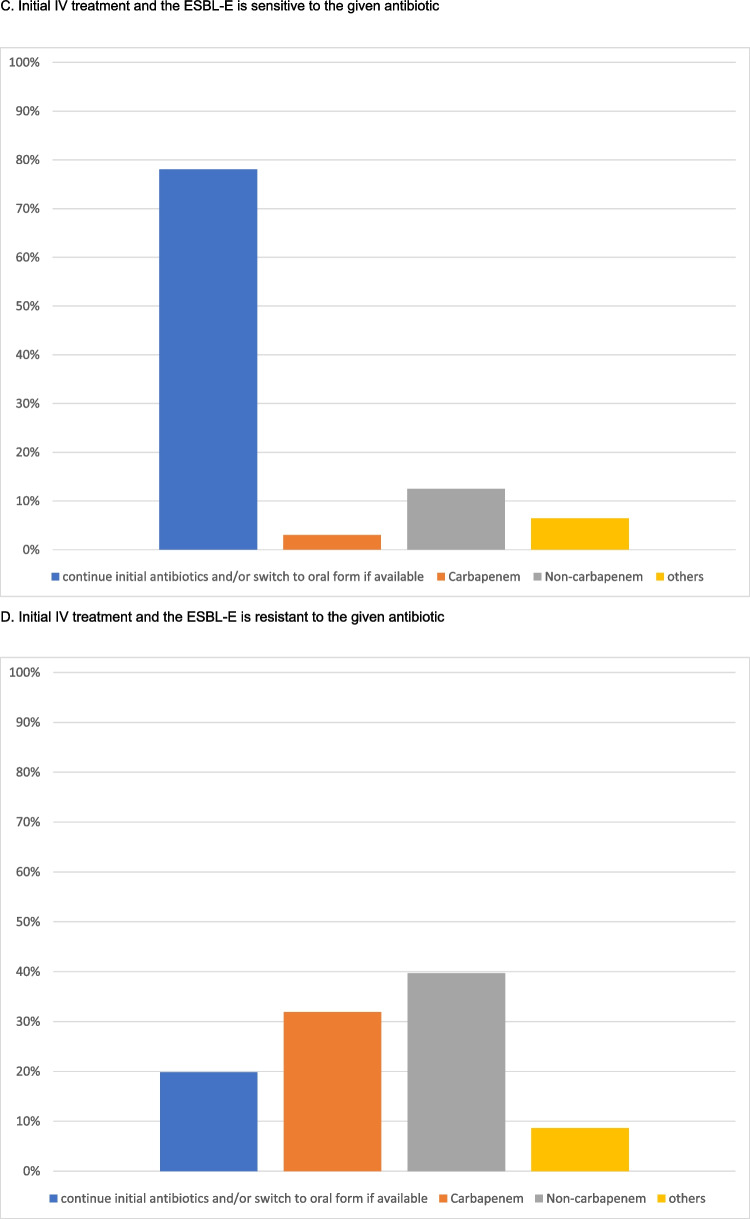
Treatment preferences in patient who showed good clinical response to the initial antibiotic, but the urine culture showed ESBL-E

Similar results were observed among centres where patients were treated with intravenous antibiotics at presentation. Most respondents (*n* = 181; 78.0%) would continue the initial antibiotics either through oral or intravenous routes if there was in vitro susceptibility. Only 22.0% (*n* = 51) of the respondents would adjust the antibiotic regimen. In the presence of in vitro resistance, 39.7% (*n* = 92) and 31.9% (*n* = 74) of them would change to another non-carbapenem antibiotic or to an intravenous carbapenem according to the sensitivity results, respectively. 19.8% (*n* = 46) would continue the initial IV antibiotics. The results are summarized in Fig. 1A–D.

We further compared the perceived efficacy and recurrence risk between carbapenems and initial non-carbapenem antibiotics by which patients showed good clinical responses. Among respondents opting for penicillin groups with beta-lactamase inhibitors, 57.8% (*n* = 26/45) and 62.2% (*n* = 28/45) either felt neutral or disagreed with the notion that carbapenem group antibiotics were associated with better efficacy and lower recurrence risk, respectively. As for those using other susceptible antibiotics, 54.8% (*n* = 17/31) and 64.5% (*n* = 20/31) were either neutral or disagreed that carbapenem was associated with better efficacy and lower recurrence risk, respectively.

ESBL-E bacteria are conventionally considered resistant to second- and third-generation cephalosporins. Among 148 respondents who opted to continue these agents as initial choice of antibiotics, 56.1% (*n* = 83 /148) stayed neutral or did not agree that changing to carbapenem antibiotics would reduce UTI recurrence risk. 52.7% (*n* = 78/148) were neutral or did not agree that changing to other non-carbapenem antibiotics was associated with lower recurrence risk. These indicated that there was similar perceived recurrence risk between cephalosporin, carbapenem and other non-carbapenem group antibiotics.

### Influence of socioeconomical status and geographical locations on treatment practice

**Fig. 2:**
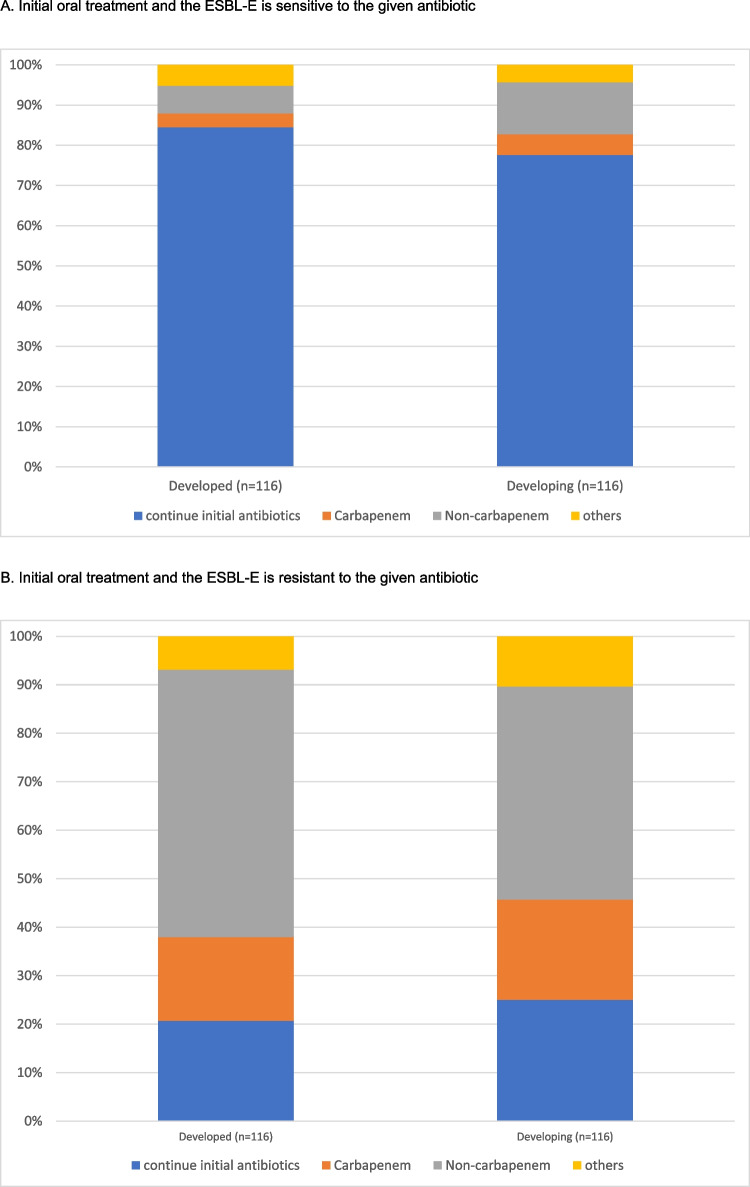

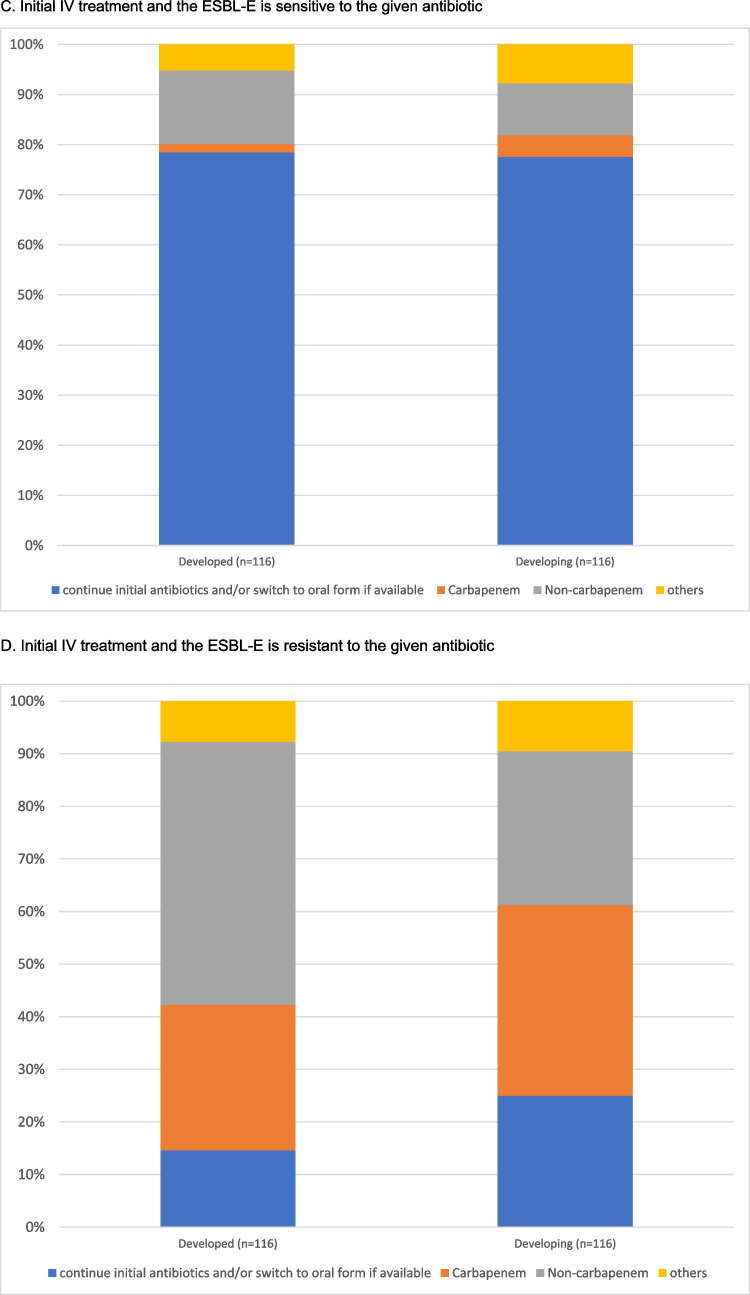
Treatment given by centres from developed and developing countries when the patient shows a good clinical response to the initial antibiotic but the culture result came back ESBL-E

**Fig. 3:**
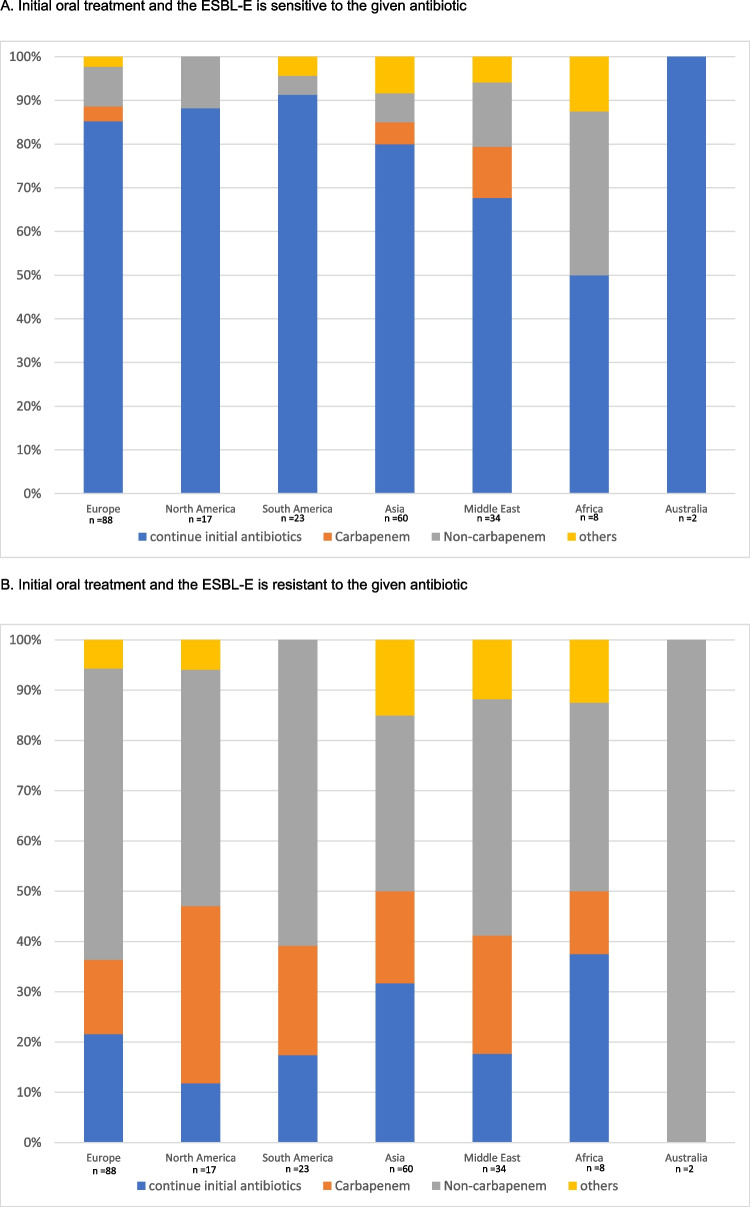

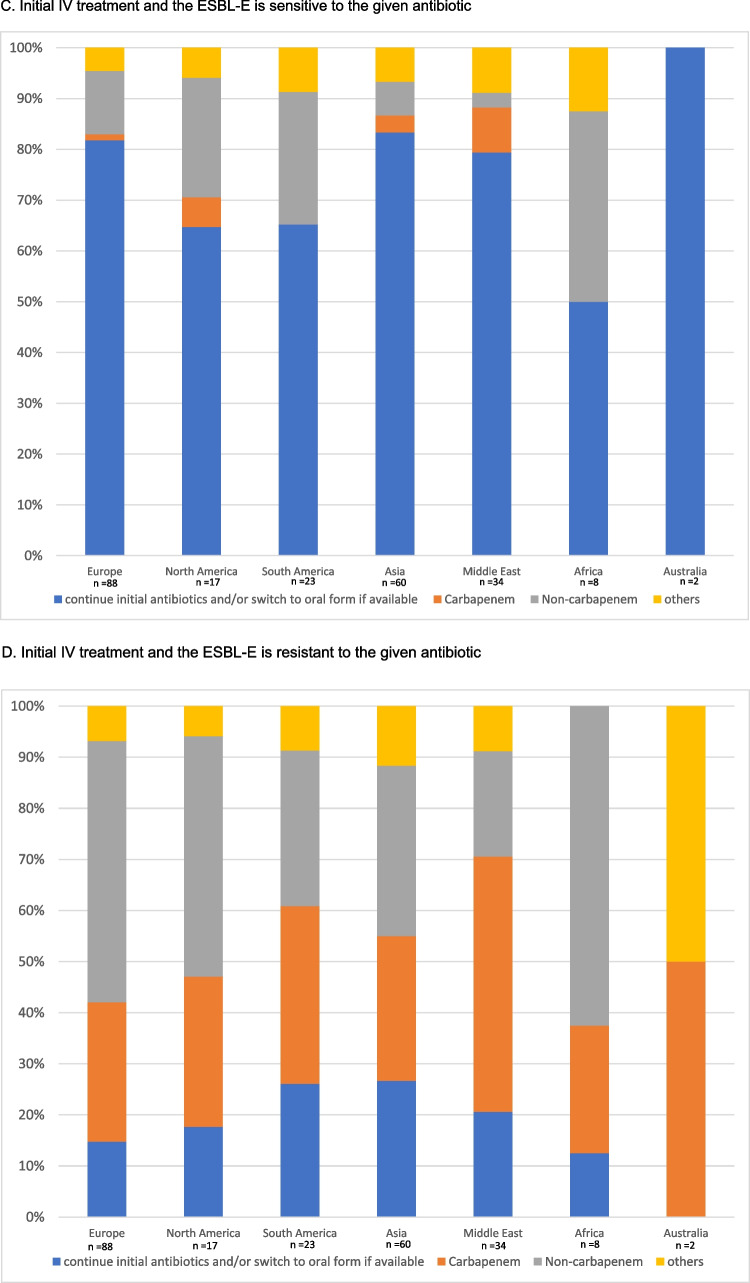
Treatment adopted by different regions when patient shows good clinical response to initial antibiotics but culture results came back ESBL E

We analysed the differences in treatment preferences by stratifying centres according to socioeconomic status and their geographic locations. Similar patterns of practice were observed between developed and developing countries (Figs. 2A–D and 3A–D). The subsequent antibiotic used would be largely guided by the in vitro susceptibility. We further evaluated the practice variations among countries with a high number of participating centres (more than 10 per country) (Supplemental Fig. 1A–D). This demonstrated a similar practice within and between countries, with the majority of the centres continuing susceptible antibiotics, and changing to appropriate antibiotics if there was in vitro resistance to initial antimicrobials.

### Follow-up management for UTI caused by ESBL-E bacteria

61.2% (*n* = 142) of the respondents would consider continuing antibiotic prophylaxis to prevent recurrent UTI caused by ESBL-E organisms (nitrofurantoin, *n* = 68/142, 47.9%; co-trimoxazole, *n* = 50/142, 35.2%; other agents, *n* = 24/142, 16.9%). The recommendation for antibiotic prophylaxis was similar between developed and developing countries (*p* = 0.50).

For follow-up imaging, 50.0% (*n* = 116) of the centres would perform radiological investigations in addition to ultrasonography of the urinary tract. Among them, 17.2% (*n* = 20/116) would perform both a micturating cystourethrogram (MCUG) and a DMSA scan, while 46.6% (*n* = 54/116) and 36.2% (*n* = 42/116) would perform either a MCUG or a DMSA scan alone. No differences in follow-up imaging practices were observed between centres from areas of different socioeconomic status (*p* = 0.28).

## Discussion

Our survey demonstrates considerable variations in the management of infants with UTI caused by ESBL-E bacteria who had responded clinically to the initial treatment. The in vitro susceptibility to the antibiotics given appeared to be of major importance in the subsequent management, with most centres switching the regimen if the bacterium was reported to be resistant to the initial antibiotic. Furthermore, the non-carbapenem group of antibiotics was perceived to be comparable to an intravenous carbapenem. Our findings suggest that there are valid carbapenem-sparing strategies to treat UTI due to ESBL-E bacteria.

Half of the participating centres reported that the incidence of infantile UTI caused by ESBL-E bacteria was over 10%, suggesting that antibiotic resistance is already a significant and increasingly common clinical challenge. Second- and third-generation cephalosporins were the initial antibiotics of choice in over 60% of respondents, yet ESBL-E bacteria are resistant to these antibiotics in vitro. The use of a cephalosporin creates selection pressure and promotes the spread of clones with ESBL activity, leading to a rising incidence of UTI caused by ESBL-E organisms [24]. Therefore, a penicillin with a beta-lactamase inhibitor, such as amoxicillin-clavulanate, may be a viable option for initial treatment in centres with a high incidence of ESBL-E infections.

In our study, the decision on the subsequent antibiotic regimen among the patients who had responded favourably to the initial treatment, was largely based on the antibiotic sensitivity profiles. Similar observations were noted in subgroup analyses among centres coming from different geographical regions and socioeconomic status. Most respondents would consider it appropriate to continue the initial antibiotics, either through oral or intravenous route, if there was in vitro susceptibility. Although the practice was more heterogeneous in the presence of in vitro resistance to the initial therapy, most centres would adjust the antibiotic regimen based on culture results. Interestingly, up to 49.6% of respondents would change to a susceptible non-carbapenem antibiotic, instead of a carbapenem. Therefore, our data do suggest that a majority of centres no longer consider changing antibiotics to the previously “standard” intravenous carbapenem to be necessary. Indeed, this preference is now supported by several more recent studies.

In a retrospective study evaluating 150 adults with acute pyelonephritis caused by ESBL-E bacteria, 56% and 44% patients had received a carbapenem or a drug from non-carbapenem-groups as initial therapy, respectively. Similar treatment response and relapse rates were observed between these two treatment arms [15]. In another retrospective multi-centre study including 492 adults suffering from UTI due to ESBL-E organisms, 65% and 35% received a carbapenem and a susceptible non-carbapenem antibiotic, respectively. Both groups achieved a high clinical response rate (97% vs. 96%, *p* = 0.28). Moreover, the microbiological eradication rate in the non-carbapenem group was statistically higher than that in the carbapenem group (98% vs. 92%, *p* < 0.05). Subgroup analysis in patients with complicated UTIs from ESBL-E organisms also revealed similar results [25]. Furthermore, in a retrospective review of 180 patients, Tullos et al. demonstrated similar efficacy between antibiotics of the carbapenem group and piperacillin-tazobactam in patients suffering from UTI due to ESBL-E bacteria susceptible to the given antibiotic [26]. Satisfactory efficacy in terms of clinical response and relapse rates was also observed among 43 children with UTI due to ESBL-E bacteria treated with a non-carbapenem antibiotic [18]. Madhi et al. reported that amikacin, an aminoglycoside, had comparable efficacy to a carbapenem in paediatric patients, and it was also associated with shorter hospital stay [16]. Non-carbapenem antibiotics, such as amoxicillin-clavulanate and fluroquinolones, are available in oral preparations that can facilitate early hospital discharge and alleviate the repeated need of vascular access and can therefore be a viable option for antibiotic switch therapy.

A small proportion of respondents would consider continuing the initial antibiotic that had given a good clinical response despite in vitro resistance. There are scarce data pertaining to this clinical decision. A previous report found that 80% of infants suffering from UTI caused by ESBL-E bacteria achieved defervescence within 3 days of monotherapy with ceftriaxone, a third-generation cephalosporin [20]. Hyun et al. reviewed the outcome of 845 paediatric patients with UTI, of whom 146 suffered from an ESBL-E infection [19]. Among them, 109 had received an initial antibiotic to which their bacteria were not susceptible. Seventy children continued the given antibiotic due to good clinical response, while only 49 changed to an antibiotic to which the bacteria were susceptible. Interestingly, the relapse rates were similar between the two groups of children, at 2.3% and 3.3%, respectively. These findings suggest that when the given antibiotic is clinically effective, the relapse rate remains low despite a lack of demonstrated in vitro susceptibility.

Clinical response to an antibiotic depends on the characteristics of the specific ESBL-E variants, the pharmacokinetics of the antibiotic and the minimal inhibitory concentration (MIC). These important pieces of information were often not given in the aforementioned studies, nor are they typically available in standard clinical situations [15–20]. More than 10 additional families of ESBL-E variants have been identified since the 1980s [27, 28]. Even within a single family, there are subtypes associated with heterogeneous clinical behaviours and different abilities to hydrolyse penicillin, cephalosporins and aztreonam [12]. For instance, the OXA-10 variant is weak in hydrolysing third-generation cephalosporins, when compared to other variants like OXA-11 and OXA-16 [27, 28]. Variable susceptibility to beta-lactamase inhibitors was similarly reported between families [28–31]. MIC is the minimal antibiotic concentration that completely prevents visible growth of an organism in a strictly controlled in vitro environment [32]. There is a close association between treatment success and the MIC of a cephalosporin in treating ESBL-E infections [33]. Specifically, treatment success was 81% at a MIC of < 1 mg/L, compared to 26% at a MIC of 4 mg/L. Pharmacokinetics is also an important consideration. For instance, amoxicillin-clavulanate is excreted into urine with 50–85% amoxicillin and 25–40% clavulanate remaining unchanged. However, only 33–67% of ceftriaxone is excreted into urine as intact drug [34]. The former can therefore achieve a higher urinary concentration. Indeed, amoxicillin-clavulanate was demonstrated to achieve and maintain its urinary concentration three-folds above the effective MIC for the majority of time between dosing interval [35, 36]. The important conclusion is that a good clinical response can be obtained in spite of antibiotic resistance as defined by routine in vitro susceptibility testing [37].

Ultrasonography is the only recommended imaging for most infants presenting with their first febrile UTI. Further investigations such as MCUG and DMSA scans should be performed only in selected patients with atypical features or recurrent infections [38]. The optimal approach for those with infections caused by ESBL-E remains unclear. Fifty-eight percent of the respondents considered undiagnosed underlying structural anomalies as a risk factor for ESBL-E infection. Previous studies have reported that VUR of any grade is associated with a three-to-eight times increased risk of developing ESBL-E infection [39–41]. The presence of ESBL-E, however, may not necessarily indicate underlying significant urological abnormalities. In a retrospective study, a similar prevalence of UTI caused by ESBL-E bacteria was observed in patients with or without urological abnormalities [42]. Indeed, the presentation of UTI caused by ESBL-E bacteria was reported to be related to local antibiotic practice, rather than an underlying urological anomaly [43]. Consequently, only half of the respondents in our survey offered additional radiological investigations.

Current guidelines recommend against antibiotic prophylaxis in children following first febrile UTI. It may be considered in children with significant urological abnormalities, such as high-grade VUR or recurrent infections. Interestingly, about 60% of respondents would consider antibiotic prophylaxis after UTI due to ESBL-E bacteria, potentially due to a concern about underlying urological abnormalities, or in the presence of bladder bowel dysfunction or frequent UTI. The role of antibiotic prophylaxis is still controversial with studies showing conflicting results [44–47]. In the RIVUR trial, which included 607 children with grade I–IV VUR (of whom 280 had grade III–IV VUR), the benefit of antibiotic prophylaxis was not demonstrated. Similar rates of developing renal scarring were observed in children with or without prophylaxis [44]. In a more recent multi-centre randomized control trial including 292 patients with grade III–V VUR, Morello et al. showed similar rates of UTI requiring hospitalization and new renal scarring between children with and without prophylaxis [48]. However, in the Swedish Reflux Trial, which recruited 203 children with grade III–IV VUR, the rate of new renal scarring following UTI was significantly lower in those on prophylaxis compared to those on surveillance [47]. Its effect in children who have had a UTI caused by ESBL-E remains even more unclear.

Our survey has a good international representation, providing practice variance across more than 200 centres worldwide. However, there are several limitations. First, the design of this work is a survey, which is opinion-based and lacks patient-level data. Furthermore, practice differences even within the same centre are common, so responses may not reflect consistent centre practices. Thirdly, we did not collect the information pertaining to the different subtypes of ESBL-E, which may have impacts on treatment response. Lastly, we did not enquire about the training status and level of care of the respondents in the initial survey. Despite an effort for a follow-up enquiry, only about half of the respondents replied and most of them were fully trained nephrologists working in tertiary centres.

In conclusion, the optimal treatment of infants with UTI caused by ESBL-E bacteria remains controversial. However, empirically switching the antibiotic to an intravenous carbapenem in patients showing a good clinical response does not appear to be the preferred management among most participating centres. Alternative approaches, such as continuing the initial antibiotics or switching to non-carbapenem antibiotics, can be viable options that may shorten hospital stay and reduce stress of the patient and carers. Future randomized clinical trials, or well-designed retrospective studies, may help to identify the optimal antibiotic regimen in this specific patient population.

## Supplementary Information

Below is the link to the electronic supplementary material.

Graphical abstract (PPTX 129 KB)

Supplementary file2 (DOCX 102 KB)

## Data Availability

All data generated or analysed during this study are included in this published article and its supplementary information files.
