# Supplementary material for: Controversies in treating febrile infantile urinary tract infection caused by extended-spectrum beta-lactamase producing Enterobacteriaceae: an international multi-centre survey
Source: Pediatr Nephrol. 2025 Feb 4;40(7):2253–66. doi: 10.1007/s00467-025-06700-w (PMC12116987; doi:10.1007/s00467-025-06700-w)
Supplement: Supplementary file 1 — Graphical abstract (PPTX 129 KB) [file 467_2025_6700_MOESM1_ESM.pptx]

## Slide 1
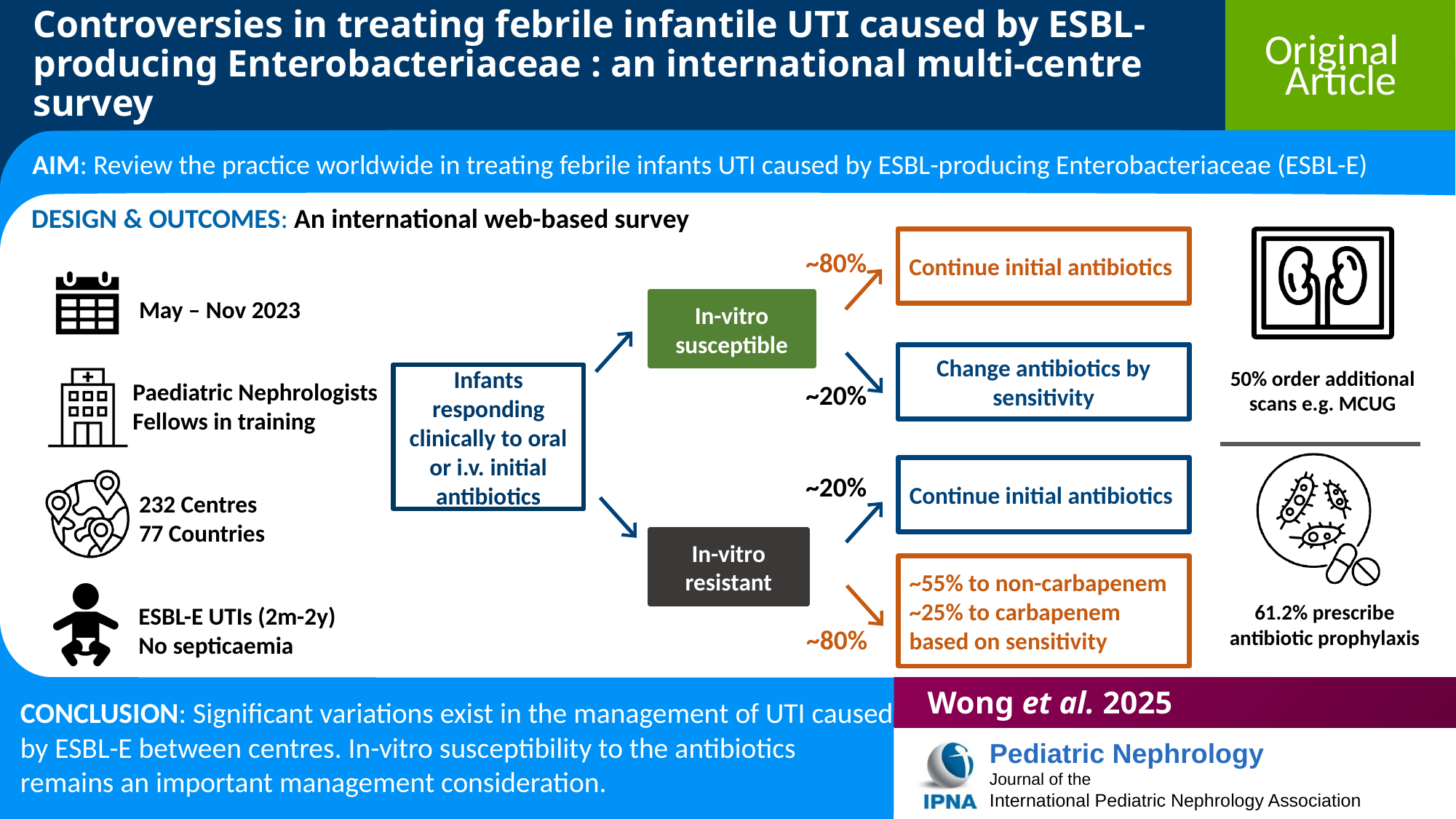

Controversies in treating febrile infantile UTI caused by ESBL-producing Enterobacteriaceae : an international multi-centre survey
AIM: Review the practice worldwide in treating febrile infants UTI caused by ESBL-producing Enterobacteriaceae (ESBL-E)
DESIGN & OUTCOMES: An international web-based survey
Continue initial antibiotics
~80%
May – Nov 2023
In-vitro susceptible
Change antibiotics by sensitivity
50% order additional scans e.g. MCUG
Infants responding clinically to oral or i.v. initial antibiotics
Paediatric Nephrologists
Fellows in training
~20%
Continue initial antibiotics
~20%
232 Centres
77 Countries
In-vitro resistant
~55% to non-carbapenem ~25% to carbapenem
based on sensitivity
61.2% prescribe antibiotic prophylaxis
ESBL-E UTIs (2m-2y)
No septicaemia
~80%
Wong et al. 2025
CONCLUSION: Significant variations exist in the management of UTI caused by ESBL-E between centres. In-vitro susceptibility to the antibiotics remains an important management consideration.
