# Supplementary material for: Controversies in treating febrile infantile urinary tract infection caused by extended-spectrum beta-lactamase producing Enterobacteriaceae: an international multi-centre survey
Source: Pediatr Nephrol. 2025 Feb 4;40(7):2253–66. doi: 10.1007/s00467-025-06700-w (PMC12116987; doi:10.1007/s00467-025-06700-w)
Supplement: Supplementary file 2 — Supplementary file2 (DOCX 102 KB) [file 467_2025_6700_MOESM2_ESM.docx]

Supplemental Materials

Form 1. Questionnaire

**How do we treat infants (2month – 2 years) with a febrile UTI that has responded to the given oral antibiotic despite having an infection with a multi-resistant ESBL producing bacteria?**

*Background:*

Antibiotic treatment of febrile UTI is in a majority of cases straightforward. The infecting strain is sensitive to the given treatment and the child responds rapidly. An important sub-group of the children is, however, infected with bacteria resistant to some or many antibiotics; some due to ESBL-producing Enterobacteriaceae (ESBL-E). UTIs caused by these latter bacteria have been increasing over the years.

The treatment of children infected with ESBL-E is more complicated. It is rather straightforward if the child has not improved with the initially given antibiotic. In those cases, intravenous carbapenem group antibiotics are often the preferred treatment. This treatment, however, leads to prolonged hospital stay, increased costs and potential problems with vascular access.

The situation becomes more complicated if the child has clinically improved despite the bacteria being defined as resistant to the initial regimen that was given either intravenously or orally.

Treatment approaches in those cases include oral switch therapy or continuing the initially given oral antibiotics. Successful responses have been reported in this group of patients. This is most likely due to high concentrations of antibiotics in the urine and the fact that antimicrobial resistance is not a yes and no phenomenon but a gradual continuum.

*Study question*

In this survey, we aim to better understand current practices and opinions among paediatric nephrologists in this controversial area.

**Name:**

**Email:**

**Center Name:**

**City/ Country:**

General information pertaining to management of first febrile UTI (2 months – 2 years)

1. Which of the following guideline(s) do you follow for treatment of febrile, infant UTI?

| □ AAP | □ NICE | □ ESPID |
| --- | --- | --- |
| □ ISDA | □ ESCMIS | □ Local guideline |
| □ Others (specify) | | |

1. What is the choice of antibiotics at first presentation?

| □ Penicillin group with Beta-lactamase inhibitors  (e.g. augmentin), | □ 2^nd^ generation cephalosporin  (e.g. cefuroxime) |
| --- | --- |
| □ Aminoglycoside  (e.g. gentamicin, amikacin | □ 3^rd^ generation cephalosporin  (e.g. cefotaxime) |
| □ Carbapenem  (e.g. meropenem) | □ Others, please specify:  _____________________ |
|  |  |

1. What is your usual initial route of administration?

| □ Intravenous (Please go to 4B) | □ Oral (Please go to 4A&B) |
| --- | --- |

1. A. Under what circumstances would you use intravenous over oral route in the initial treatment?

| □ Severe sepsis | □ Known urological abnormalities |
| --- | --- |
| □ History of ESBL+ UTI | □ Others: ____________________ |

B. Would you use the oral switch approach if patients improve?

| □ 24-48 hours of defervescence | □ >48 hours of defervescence |
| --- | --- |
| □ No | □ Others (specify) |

1. What is the total duration of treatment (IV and oral) in the absence of bacteremia?

| □ <7 days | □ 7-10 days |
| --- | --- |
| □ 11-14 days | □ >14 days |

Questions related to febrile UTI caused by ESBL-E

1. What is the perceived proportion of UTI in your center caused by ESBL-E?

| □ < 10% | □ 11-20% |
| --- | --- |
| □ 21-30% | □ > 30% |

1. What action would you take if the urine culture reveals ESBL-E but patients show good clinical response to the current antibiotics (i.e. defervescence and remains clinically well without sepsis and bacteremia)?

    Please select your response to the following scenarios:

| A. If **oral** treatment was started and the ESBL-E is **sensitive** to the antibiotics  Continue current oral antibiotics, and keep the same duration of treatment  Continue current oral antibiotics, but for a longer duration, e.g. 10-14 days  Change to intravenous carbapenem group antibiotics  Change to oral non-carbapenem group antibiotics according to culture sensitivity  Change to intravenous non-carbapenem group antibiotics according to culture sensitivity  Others, please specify __________ |
| --- |
| B. If **oral** treatment was started and the ESBL-E is **resistant** to the antibiotics  Continue current oral antibiotics, and keep the same duration of treatment  Continue current antibiotics, but for a longer duration, e.g. 10-14 days  Change to intravenous carbapenem group antibiotics  Change to oral non-carbapenem group antibiotics according to culture sensitivity  Change to intravenous non-carbapenem group antibiotics according to culture sensitivity  Others, please specify ________ |
| C. If **IV** treatment was started and the pathogen is **sensitive** to the antibiotics  Continue current IV antibiotics, and keep the same duration of treatment  Continue current IV antibiotics, but for a longer duration, e.g. 10-14 days  Switch to the oral form of the same antibiotic, and keep the same total duration of treatment  Switch to the oral form of the same antibiotic, but for a longer duration, e.g. 10-14 days  Change to intravenous carbapenem group antibiotics  Change to oral non-carbapenem group antibiotics according to culture sensitivity  Change to intravenous non-carbapenem group antibiotics according to culture sensitivity  Others, please specify ________ |
| D. If **IV** treatment was started and the pathogen is **resistant** to the antibiotics  Continue current IV antibiotics, and keep the same duration of treatment  Continue current IV antibiotics, but for a longer duration, e.g. 10-14 days  Switch to the oral form of the same antibiotic, and keep the same total duration of treatment  Switch to the oral form of the same antibiotic, but for a longer duration, e.g. 10-14 days  Change to intravenous carbapenem group antibiotics  Change to oral non-carbapenem group antibiotics according to culture sensitivity  Change to intravenous non-carbapenem group antibiotics according to culture sensitivity  Others, please specify ________ |

1. Do you agree it is reasonable to **continue current antibiotics and/or oral switch**, even if its resistant in-vitro, if the patients demonstrate good clinical response?

| Strongly disagree Disagree Neutral Agree Strongly agree |
| --- |

1. Do you agree **stepping up to carbapenem group** has the following benefits compared to continuing initial treatment in ESBL-UTI responding to treatments:

| Offer Better treatment efficacy |  |
| --- | --- |
| Strongly disagree Disagree Neutral Agree Strongly agree | |
| Less UTI recurrence risk (within 2 months) |  |
| Strongly disagree Disagree Neutral Agree Strongly agree | |

1. Do you agree **changing to non-carbapenem group** has the following benefits compared to continuing initial treatment in ESBL-UTI responding to treatments:

| Better treatment efficacy |  |
| --- | --- |
| Strongly disagree Disagree Neutral Agree Strongly agree | |
| Less recurrence risk (within 2 months) |  |
| Strongly disagree Disagree Neutral Agree Strongly agree | |

1. Do you agree **stepping up to carbapenem** has the following benefits compared to non-carbapenem in ESBL-UTI responding to treatments:

| Better treatment efficacy |  |
| --- | --- |
| Strongly disagree Disagree Neutral Agree Strongly agree | |
| Less recurrence risk (within 2 months) |  |
| Strongly disagree Disagree Neutral Agree Strongly agree | |

1. Is the presence of ESBL-producing organism an indicator for additional investigation

| Yes (more than 1 can be selected)  Please specify -  Micturating cystourethrogram  DMSA scan | No |
| --- | --- |

1. Would you change your initial UTI treatment to ESBL-E specific treatment in the following scenarios?

| 1. Known underlying urological abnormalities | |
| --- | --- |
| Yes | No |
| 1. Hospitalization within 3 months | |
| Yes | No |
| 1. Antibiotics exposure within 3 months | |
| Yes | No |

1. Would you consider prophylaxis for children with the recurrent ESBL-E UTI?

| Yes  cotrimoxazole  nitrofurantoin  Others: ____________________ | No |
| --- | --- |

1. **Are you interested in joining a retrospective study on treatment outcomes of ESBL-E UTI?**

| Yes | No |
| --- | --- |

Table 1. Distribution of Participating Centres

| Continents | | | | | | | | | | | | | |
| --- | --- | --- | --- | --- | --- | --- | --- | --- | --- | --- | --- | --- | --- |
| Europe  (n=88) |  | North America (n=17) |  | South America (N=23) |  | Asian (n=60) |  | Middle East (n=34) |  | Africa (n=8) |  | Oceania  (n=2) |  |
| Centres from Developed Countries (n=116) | | | | | | | | | | | | | |
| Austria | 1 | Canada | 3 |  |  | Hong Kong | 2 |  |  |  |  | Australia | 2 |
| Belgium | 5 | USA | 14 |  |  | Japan | 6 |  |  |  |  |  |  |
| Bulgaria | 1 |  |  |  |  | South Korea | 2 |  |  |  |  |  |  |
| Czech Republic | 2 |  |  |  |  | Taiwan | 1 |  |  |  |  |  |  |
| Croatia | 2 |  |  |  |  |  |  |  |  |  |  |  |  |
| Denmark | 2 |  |  |  |  |  |  |  |  |  |  |  |  |
| France | 5 |  |  |  |  |  |  |  |  |  |  |  |  |
| Georgia | 2 |  |  |  |  |  |  |  |  |  |  |  |  |
| Germany | 3 |  |  |  |  |  |  |  |  |  |  |  |  |
| Spain | 6 |  |  |  |  |  |  |  |  |  |  |  |  |
| Greece | 1 |  |  |  |  |  |  |  |  |  |  |  |  |
| Italy | 11 |  |  |  |  |  |  |  |  |  |  |  |  |
| Montenegro | 1 |  |  |  |  |  |  |  |  |  |  |  |  |
| Netherlands | 4 |  |  |  |  |  |  |  |  |  |  |  |  |
| North Macedonia | 2 |  |  |  |  |  |  |  |  |  |  |  |  |
| Norway | 1 |  |  |  |  |  |  |  |  |  |  |  |  |
| Poland | 8 |  |  |  |  |  |  |  |  |  |  |  |  |
| Portugal | 9 |  |  |  |  |  |  |  |  |  |  |  |  |
| Romania | 4 |  |  |  |  |  |  |  |  |  |  |  |  |
| Slovenia | 2 |  |  |  |  |  |  |  |  |  |  |  |  |
| Sweden | 5 |  |  |  |  |  |  |  |  |  |  |  |  |
| Switzerland | 3 |  |  |  |  |  |  |  |  |  |  |  |  |
| UK | 4 |  |  |  |  |  |  |  |  |  |  |  |  |
| Ukraine | 2 |  |  |  |  |  |  |  |  |  |  |  |  |
| Centres from Developing Countries (n=116) | | | | | | | | | | | | | |
| Russia | 2 |  |  | Argentina | 5 | Bangladesh | 3 | Iran | 3 | Bénin | 1 |  |  |
|  |  |  |  | Brazil | 3 | Cambodia | 1 | Jordan | 2 | Congo | 2 |  |  |
|  |  |  |  | Chile | 5 | China | 1 | Lebanon | 1 | Maroc | 1 |  |  |
|  |  |  |  | Colombia | 1 | India | 15 | Oman | 1 | South Africa | 1 |  |  |
|  |  |  |  | Ecuador | 1 | Indonesia | 2 | Saudi Arabia | 5 | Tunisia | 2 |  |  |
|  |  |  |  | Jamaica | 1 | Malaysia | 4 | Serbia | 4 | Zambia | 1 |  |  |
|  |  |  |  | MEXICO | 1 | Myanmar | 1 | Turkey | 17 |  |  |  |  |
|  |  |  |  | Paraguay | 2 | Nepal | 1 | United Arab Emirates | 1 |  |  |  |  |
|  |  |  |  | Peru | 3 | Pakistan | 3 |  |  |  |  |  |  |
|  |  |  |  | Uruguay | 1 | Philippines | 14 |  |  |  |  |  |  |
|  |  |  |  |  |  | SRI Lanka | 2 |  |  |  |  |  |  |
|  |  |  |  |  |  | Thailand | 2 |  |  |  |  |  |  |

Figure 1. Treatment adopted by countries with more than 10 participating centres when patients show good clinical response to initial antibiotics, but culture results came back ESBL E

A. Initial oral treatment and the ESBL-E is sensitive to the given antibiotic

n=14

n=17

n=15

n=14

n=14

n=17

n=15

n=14

B. Initial oral treatment and the ESBL-E is resistant to the given antibiotic

C. Initial IV treatment and the ESBL-E is sensitive to the given antibiotic

D. Initial IV treatment and the ESBL-E is resistant to the given antibiotic

n=14

n=17

n=15

n=14
